# Emerging Therapeutic Modalities against COVID-19

**DOI:** 10.3390/ph13080188

**Published:** 2020-08-08

**Authors:** Shipra Malik, Anisha Gupta, Xiaobo Zhong, Theodore P. Rasmussen, Jose E. Manautou, Raman Bahal

**Affiliations:** 1 Department of Pharmaceutical Sciences, University of Connecticut, Storrs, CT 06269, USA; shipra.malik@uconn.edu (S.M.); xiaobo.zhong@uconn.edu (X.Z.); theodore.rasmussen@uconn.edu (T.P.R.); jose.manautou@uconn.edu (J.E.M.); 2 Department of Chemistry, Wesleyan University, Middletown, CT 06459, USA; agupta01@wesleyan.edu

**Keywords:** SARS-CoV-2, coronavirus, COVID-19, remdesivir, antiviral, vaccine

## Abstract

The novel SARS-CoV-2 virus has quickly spread worldwide, bringing the whole world as well as the economy to a standstill. As the world is struggling to minimize the transmission of this devastating disease, several strategies are being actively deployed to develop therapeutic interventions. Pharmaceutical companies and academic researchers are relentlessly working to investigate experimental, repurposed or FDA-approved drugs on a compassionate basis and novel biologics for SARS-CoV-2 prophylaxis and treatment. Presently, a tremendous surge of COVID-19 clinical trials are advancing through different stages. Among currently registered clinical efforts, ~86% are centered on testing small molecules or antibodies either alone or in combination with immunomodulators. The rest ~14% of clinical efforts are aimed at evaluating vaccines and convalescent plasma-based therapies to mitigate the disease's symptoms. This review provides a comprehensive overview of current therapeutic modalities being evaluated against SARS-CoV-2 virus in clinical trials.

## 1. Introduction

Coronavirus disease 2019 or COVID-19 caused by a novel human coronavirus 2019 (HCoV-19/2019-nCoV/SARS-CoV-2), was officially declared a global pandemic by the World Health Organization (WHO) on 11 March 2020. The initial COVID-19 containing cases were first reported in the South China seafood market in Wuhan (Hubei Province, China) in December 2019 [1]. Several studies established that COVID-19 is highly contagious and rapidly transmits (a basic reproduction number (R0) of ~1.5–5.2) via respiratory droplets [2,3,4]. Further investigation also revealed that SARS-CoV-2 remains viable and virulent in aerosol form for hours and on surfaces for days [5]. The disease has spread rapidly across the globe, bringing the entire world economy to a standstill. The total number of cases of COVID-19 has exceeded ~10 million globally, causing more than ~500,000 deaths as of early July, 2020 [6]. SARS-CoV-2 was first identified in the bronchoalveolar fluid lavage samples from patients in Wuhan Jinyintan hospital on December 30, 2019 [7]. The virus sequencing results from the lavage samples and phylogenetic analyses showed an 86.9% conservation with bat SARS-like CoV (bat-SL-COVZC45, MG772933.1) and confirmed it as a novel zoonotic strain of coronavirus.

SARS-CoV-2, a β-coronavirus, belongs to one of the four genera of the Coronavirinae subfamily, namely the alpha, beta, delta, and gamma coronaviruses. Among these genera, only alpha and beta coronaviruses are known to infect mammals. There are six coronavirus species; SARS-CoV, Middle East Respiratory Syndrome (MERS) CoV, NL63, OC43, 229E, and HKU1 known to infect humans. SARS-CoV, and MERS-CoV are highly pathogenic viruses which have led to two global pandemics; the remaining four species only cause mild respiratory symptoms [8]. Further investigations showed that SARS-CoV-2 probably originated in bats and had 96.2% nucleotide sequence overlap with RatG13 [9], a bat coronavirus detected in *Rhinolophus affini*. However, based on few findings, pangolins are considered as a possible intermediate host of SARS-CoV-2 to bridge the zoonotic gap between bats and humans [10]. SARS-CoV-2 is the third reported zoonotic spillover from the coronavirus family in the last two decades that has led to a devastating global pandemic.

**Figure 1:**
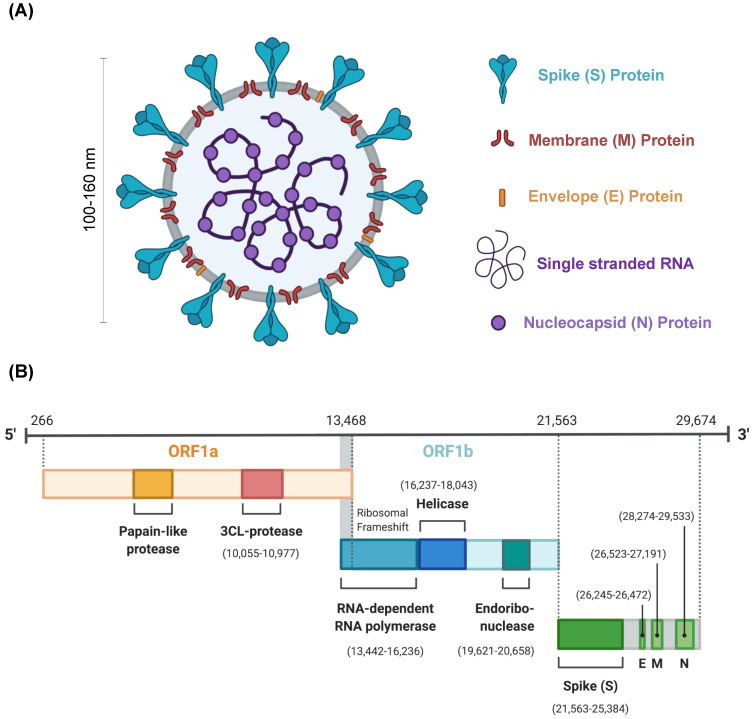
SARS-CoV-2 virus structure and genome. (**A**) Structure of SARS-CoV-2 virus including membrane (M), spike (S), envelope (E), and nucleocapsid (N) proteins, and single stranded RNA. The size of the virus is reported to be 100–160 nm. (**B**) The genomic structure of SARS-CoV-2 depicting open reading frames (ORF1a and 1b) with nonstructural proteins like 3CL protease, RNA dependent RNA polymerase (RdRp), helicase, endoribonuclease, and four structural proteins (S, M, E and N).

The SARS-CoV-2 virus has a spherical morphology with a diameter of ~100–160 nm and spikes of ~9–12 nm thickness [7]. The SARS-CoV-2 viral genome consists of a positive-sense single-stranded RNA of size 29 kilobases (kb). The single RNA strand has multiple open reading frames (ORFs) encoding for non-structural proteins, while genes which encode structural proteins are scattered between them. The SARS-CoV-2 contains nucleocapsid protein (N), spike glycoprotein (S), membrane glycoprotein (M), envelope glycoprotein (E) (Figure 1) as structural components.

The S glycoprotein of SARS-CoV-2 has been found to recognize the angiotensin-converting enzyme-2 (ACE-2) present on corneal, intestinal, nasal, and bronchial epithelium cells [11], liver cholangiocytes [12], kidney proximal tubules [13] and pneumocytes [14,15]. It has been established in multiple studies that SARS-CoV-2 utilizes the ACE2-based pathway to intrude the host cells [7,16,17,18]. Interestingly the spike protein of SARS-CoV-2 shows less than 75% sequence identity with SARS-CoV. The spike protein (S) has two sub-units: S1 and S2. S1 is the top bulb region that interacts with the ACE2 receptor and S2 is the base stalk that assists in the fusion of the viral envelope with the host cell prior to release of the nucleocapsid [19]. The interaction mentioned above between the spike protein and the host receptor is responsible for cross-species and human-to-human transmission. The activation of spike protein on interaction with ACE2 is mediated by host proteases, cathepsin B/L and a transmembrane protease serine type 2 (TMPRSS2), which cleaves the spike protein into S1 and S2 subunits. The receptor-binding motif (RBM) in the receptor-binding domain (RBD) of the S1 subunit interacts with the ACE2 receptor to induce a conformational change in S2 subunit from a pre-fusion to a post-fusion state, allowing the fusion peptide to insert into the host cell membrane [20]. A recent study revealed that SARS-CoV-2 contains a polybasic (furin) cleavage site between S1 and S2 subunits, which is different from SARS-CoV and might contribute towards its tropism and high pathogenicity [17]. In the host cell, ORF1a and ORF1b undergo translation using host machinery to generate polyprotein (pp) 1a and 1ab [19]. Next, pp1a and pp1ab undergo auto-proteolytic cleavage, resulting in 15–16 non-structural proteins with specific functions. The viral enzyme, RNA dependent RNA polymerase (RdRp) mediates the synthesis of negative sense viral genome that acts as template for the production of new viral RNAs. Further, genes encoding the structural proteins undergo translation in the endoplasmic reticulum (ER). All the viral structural components (S, M, N, and E proteins), including the positive-sense RNA, are assembled as virion particles in ER-Golgi complexes and released from cells by exocytosis [21].

## 2. Clinical Approaches

To date, no FDA-approved drug or vaccine is available to combat the COVID-19. However, comprehensive research studies are in process globally to find treatments for asymptomatic and symptomatic cases of COVID-19. Strides have been made to determine the SARS-CoV-2 origin, genome sequence, similarities with existing SARS-CoV viruses, and structural features of the spike protein, in hopes of identifying novel targets followed by effective new therapeutic interventions on time to reduce transmission as well as mortality associated with COVID-19. Given the knowledge and outcomes from previous pandemics: SARS in 2002–2004, MERS in 2012, and Ebola in 2014–2016, in combination with scientific advancements and interdisciplinary research efforts, tremendous progress has been made in evaluating multiple strategies to treat SARS-CoV-2 viral infection. Hence to find the most effective SARS-CoV-2 therapy, drugs are under development to target various phases of viral life cycle; adhesion and viral entry to host cell, endocytosis, replication, viral protease, inhibition of cytokine storm and to reduce the freely circulating viral payload [22]. 

**Figure 2:**
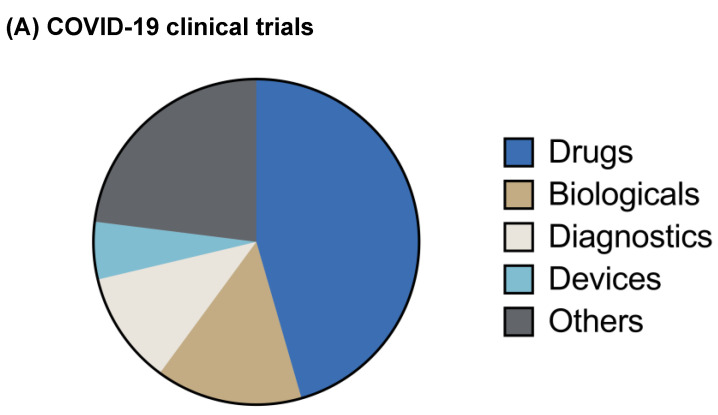

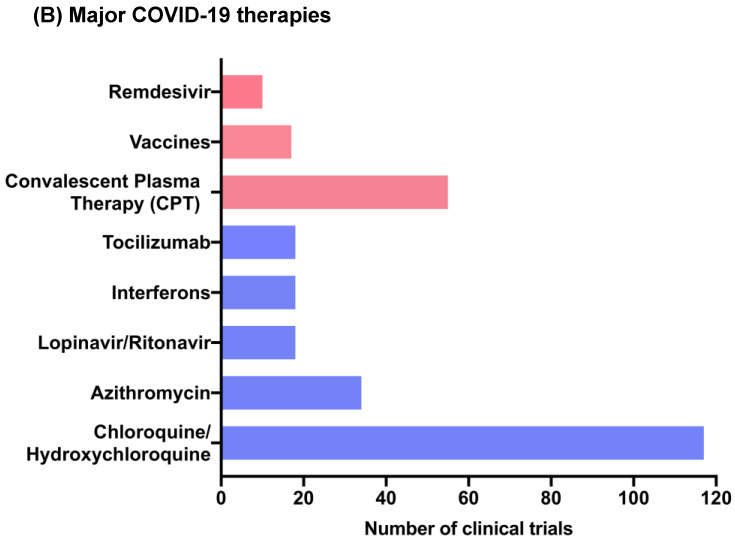
Clinical trials for COVID-19 treatment. (**A**) Distribution of COVID-19 clinical trials. Twelve hundred and sixty-five clinical trials are registered for COVID-19, which can be classified into drugs, biologicals, diagnostic tests, devices (respiratory, oxygen therapy, etc), and others (procedures, dietary supplements, etc). Four hundred and forty-seven drug candidates including repurposed and investigational are currently being tested at different stages of clinical trials for COVID-19 treatment. Furthermore, the efficacy of one hundred and forty-three biologicals including vaccines, convalescent plasma therapy, monoclonal antibodies, and stem cells is also being tested for COVID-19. (**B**) Graphical representation of major therapies against COVID-19 based on number of clinical trials. Chloroquine/hydroxychloroquine is being evaluated in one hundred and seventeen clinical trials for COVID-19 either alone or in combination with additional antiviral or immunomodulatory agents. Convalescent plasma is another major investigational therapy whose efficacy is now being evaluated in fifty-five clinical trials for COVID-19 treatment.

Approximately 1265 clinical trials related to COVID-19 are registered [23], including diagnostic tests, devices, and treatment strategies against SARS-CoV-2 (Figure 2A). Some 447 clinical trials are ongoing to evaluate novel therapeutic modalities, repurposed antivirals (lopinavir/ritonavir/ ribavirin), antimalarial (hydroxychloroquine), and anti-inflammatory drugs for treating SARS-CoV-2 infection. Also, 143 biologicals are at different stages of clinical trials, including seven vaccine candidates (Figure 2B). This review is centered on providing an inclusive update on the aforementioned therapeutic interventions for treating COVID-19.

### 2.1. Repurposed Drugs

Drug repurposing (or re-tasking, reprofiling, or repositioning) is an approach to identify new therapeutic uses of approved drugs [24]. The development of new antiviral drugs against SARS-CoV-2 and their utility in the clinic will require years of extensive research in addition to lengthy administrative approval processes. Due to the high transmission rate and mortality associated with SARS-CoV-2 infection, the hunt for an approved drug that can be repurposed to target SARS-CoV-2 is ongoing.

To screen potential candidates for repurposing, Gordon et al. utilized affinity mass spectroscopy to identify multiple SARS-CoV-2 as well as human protein-protein interaction (PPI) targets involved in various stages of viral replication and pathogenesis [25]. Based on chemoinformatic analyses, 66 human target proteins have been identified from SARS-CoV-2-human interactome. These proteins can act as a promising molecular target using the repurposed drugs-based strategy at different stages of clinical or pre-clinical development of SARS-CoV-2 treatment.

The majority of repurposed drugs, as discussed below and summarized in Table 1, are in clinical trials to find effective treatment against COVID-19. If successful, repurposed medicines will save significant time and resources while making the drugs accessible to the SARS-CoV-2 infected patients across the globe.

#### 2.1.1. Lopinavir/Ritonavir

**Figure 3:**
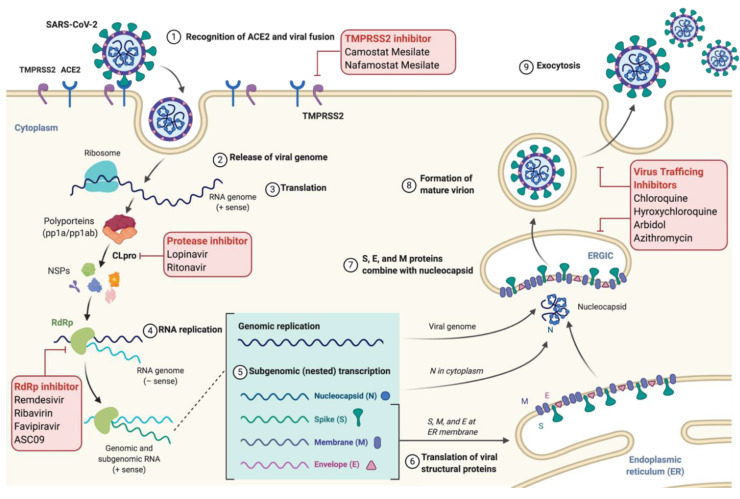
Graphical abstract depicting the SARS-CoV-2 replication cycle and site of action for widely studied drugs against COVID-19. (1) The virus enters the host cell by recognizing the ACE2 receptor via spike glycoprotein which induces membrane fusion, resulting in (2) release of viral genome in the cytoplasm. (3) The viral RNA undergoes translation to form polyproteins, which are then cleaved by the viral protease enzyme (CLpro) to form nonstructural proteins like RdRp for replication of viral RNA. Positive sense of viral RNA then undergoes translation to form structural proteins (N, S, M, and E) where S, M, and E are processed in ER (6), while N protein is processed in the cytoplasm where it assembles with a viral RNA replicon. All components are then combined inside the ER-golgi intercompartment (ERGIC) (7), from which virions are released inside the vesicles (8) and secreted outside the cell via exocytosis (9). ACE2: Angiotensin Converting Enzyme 2; TMPRSS2: Type 2 Transmembrane Serine Protease; NSPs: Non-structural proteins; RdRp: RNA dependent RNA polymerase; and CLPro: Coronavirus Protease.

The combination of anti-retroviral drugs, lopinavir and ritonavir (LPVr), is approved by the FDA (Kaletra®) for human immunodeficiency virus (HIV)-1 treatment in adults and pediatric patients since 2000 [26]. Lopinavir and ritonavir are potent inhibitors of HIV-1 protease (Figure 3). Ritonavir is a cytochrome P450 inhibitor that increases the bioavailability of lopinavir when administered in combination. The coronavirus protease (CLpro) cleaves the polyproteins into non-structural proteins essential for viral replication, hence makes it an attractive target for antiviral therapy [58]. Lopinavir was tested against SARS-CoV in multiple in vitro studies [59,60] and also reported to improve the clinical outcome of SARS-CoV infected patients in a retrospective cohort study [61]. Similarly, lopinavir showed optimal activity against MERS-CoV in vitro [62] as well as in vivo [63]. In an in vitro study, an EC_50_ of 26.63 μM was reported for lopinavir against SARS-CoV-2, which is significantly lower than the observed serum concentrations achieved with clinical doses of lopinavir/ritonavir combination (100–400 mg twice daily) [64]. However, the results of the first clinical trial of LPVr (100–400 mg twice a day for 14 days) in severe COVID-19 infected patients (ChiCTR2000029308) showed no clinical improvement in comparison to the standard care [65]. Similarly, the results from another clinical trial (NCT0425885, ELACOI) also found little or no benefit of LPVr over the control group [27]. The HIV-1 protease belongs to aspartic acid protease family and LPVr fits in the C2 symmetry region of the catalytic site of the protease and inhibits its activity. The SARS-CoV-2 protease, CLpro, lacks the specific binding site for LPVr leading to its poor activity [66].

Moreover, two large scale clinical trials including RECOVERY and SOLIDARITY-WHO discontinued their LPVr study arm based on preliminary results which showed no potential benefits of using LPVr in comparison to the standard care. Presumably, the results from on-going clinical trials (18) centered on dose optimization and treatment duration will provide more insights into the clinical efficacy of LPVr against SARS-CoV-2.

#### 2.1.2. Chloroquine/Hydroxychloroquine

Chloroquine (CQ), an antimalarial and amoebicidal drug, was first approved by the FDA in 1949 [67]. A derivative of CQ; hydroxychloroquine (HCQ) shows a superior safety profile and is approved for treatment of rheumatoid arthritis, malaria and systemic lupus erythematosus [68]. Mechanistically, CQ and HCQ acts by inhibiting the heme polymerase enzyme in trophozoites resulting in accumulation of toxic heme that causes parasite death [69]. As a repurposed drug, the antiviral activities of CQ have been reported against multiple coronavirus strains, including HCoV-229E [70], SARS-CoV [71], and MERS-CoV [62] in vitro. A study in pregnant mice infected with HCoV-O43 and treated with CQ (5 mg/kg), reported 88% survival rate for newborn mice in comparison to 20% survival rate of untreated mice six days post infection [72]. Similarly, a recent study confirmed the EC_50_ of 1.13 μM and EC_90_ of 6.9 μM for CQ against SARS-CoV-2 in vitro [73]. The antiviral activity of CQ and HCQ was compared in vitro and CQ was found to be more effective at a low dose (EC_50_ = 2.71 μM) than HCQ (EC_50_ = 4.51 μM) [74]. The antiviral activity of CQ is due to acidification of endosomes/lysosomes that interferes with fusion, replication, and release of the SARS-CoV virus from the host cells [75,76]. Besides, CQ is known to impair the glycosylation of ACE2 enzyme, which further prevents the entry of virus in the host cells [71]. Based on these promising studies, the efficacy of HCQ and CQ is being evaluated in 117 clinical trials for COVID-19. The preliminary results from a clinical trial in 100 patients indicated the optimal efficacy of CQ in comparison to the control group [77]. Similarly, HCQ was reported to be partially effective in a clinical trial conducted in 62 patients, where 80.6% of patients showed improvement in pneumonia [78]. On March 28, 2020, the FDA granted emergency use authorization (EUA) to hydroxychloroquine sulfate and chloroquine phosphate to treat hospitalized patients of COVID-19. A later clinical trial with 368 COVID-19 patients in US veterans’ hospitals concluded that use of HCQ either with or without azithromycin did not reduce the risk of mechanical ventilation, but rather increased overall mortality [79].

However, recent results from a clinical trial (NCT04323527) that evaluated clinical safety of CQ at a lower (total 2.7 g CQ in 10 days) and a higher dose (total 12 g CQ in 10 days) in COVID-19 patients noticed QT-interval prolongation (a heart rhythm abnormality) and high mortality rate (39%) in the higher dose arm of the trial [80]. Recently, multiple observational studies reported QT prolongation on use of HCQ or CQ in COVID-19 patients, and combination with azithromycin increased the risk of sudden cardiac death and severe QT prolongation [81,82]. Recently, three large scale clinical trials evaluating the efficacy of HCQ for COVID-19, including RECOVERY, SOLIDARITY-WHO, or ORCHID-NIH were suspended due to lack of efficacy in preliminary results [28]. Hence, based on the new evidence, FDA revoked the EUA on June 15, 2020 as results from multiple studies failed to prove the potential efficacy of HCQ for treatment of COVID-19. Further results from another clinical trial (NCT04322123) conducted in 667 patients reported that HCQ with or without azithromycin did not improve the clinical status of COVID-19 patients after 15 days in comparison to standard care and resulted in prolongation of QT interval [29]. Additional concerns have been raised regarding bias in the reported studies and papers have been retracted from prestigious journals [30]. Hence, complete results from on-going clinical trials are essential to establish the clinical efficacy and toxic effects of HCQ in COVID-19 patients. 

#### 2.1.3. Azithromycin

Azithromycin is a broad-spectrum antibiotic and is used for the treatment of myriads of bacterial infections like sinusitis, pneumonia, pharyngitis, urethritis, and cervicitis. Azithromycin binds with the 50S ribosomal subunit of 23S rRNA and results in the inhibition of bacterial protein synthesis [83]. Interestingly, azithromycin shows antiviral effect against Zika, Rhinovirus, Ebola, and H1N1 influenza viruses in vitro [84]. In line with this, azithromycin demonstrated an EC50 of 2.12 μM against SARS-CoV-2 in vitro [85]. The synergistic effect of azithromycin (10 μM) was assessed in combination with HCQ (2 μM) in vero E6 cells to inhibit SARS-CoV-2 replication [31]. Although a precise antiviral mechanism of azithromycin still needs to be explored, it is plausible that the accumulation results in endosome acidification that further blocks the viral genome's release. In addition, azithromycin induces anti-inflammatory response by inhibition of STAT1 and NF-κB pathways [32] and protects against Zika virus infection via interferon upregulation [86].

Azithromycin was also used as an adjuvant therapy in combination with HCQ in a non-randomized clinical trial to prevent secondary bacterial infections in COVID-19 patients [87]. Also, the safety and efficacy of azithromycin, combined with HCQ are being evaluated in 34 clinical trials. Preliminary results have raised concerns regarding the use of azithromycin in combination with HCQ due to severe QT prolongation and ventricular arrhythmia noted during clinical trial studies [81,82,88].

#### 2.1.4. Arbidol

Arbidol (ARB), also known as umifenovir, is a broad-spectrum antiviral drug [89]. ARB is an indole derivative and is commercially available in Russia and China for the treatment of influenza and respiratory viral infections [90]. ARB is effective against a range of viruses: Ebola, poliovirus type 3, herpes, and Tacaribe virus both in vitro [91] and in vivo [90] at micromolar concentrations. A recent study reported an EC50 of 4.11 μM for ARB against SARS-CoV-2, which can be achieved in serum at a clinically approved dose (200 mg, three times a day) [92]. ARB acts directly on the virus and also inhibits the viral replication indirectly via host; hence the dual mechanism contributes to its broad-spectrum antiviral activity. Multiple mechanisms have been proposed for ARB, including interaction with aromatic residues in the viral glycoprotein to prevent fusion with the host cell [93], interference with viral intracellular trafficking [94], and stabilization of influenza virus hemagglutinin (HA) to inhibit confirmation change necessary for virus-host fusion [95]. In a recent simulation study, ARB was reported to block the trimerization of the spike glycoprotein (S) of SARS-CoV-2, required for fusion with host ACE2 receptor and leads to the less virulent form of SARS-CoV-2 [33].

Currently, three clinical trials centered on ARB treatment for COVID-19 are ongoing. In a randomized controlled study, ARB monotherapy (*n* = 35) showed slight activity as compared to the standard supportive care (*n* = 17) in COVID-19 patients [27]. On the contrary, a retrospective cohort study demonstrated the improvement in viral clearance in ARB treated group (*n* = 49) [96]. Another study determined that the combination of LPVr and ARB could be beneficial in the early stages of the infection as compared to monotherapies [97]. The safety and efficacy of ARB were also compared against LPVr in COVID-19 patients, where ARB treated group (*n* = 16) showed no viral loads after 14 days of treatment, however, 44.1% of patients in LPVr group (*n* = 32) tested positive [34]. Although the results are promising for combination and monotherapy, caution needs to be exercised for its broad clinical application as design and sample size of the studies are not robust. This highlights the need for large-scale randomized clinical trials.

#### 2.1.5. Serine Protease Inhibitors

Camostat mesylate (Foipan®), a serine protease inhibitor, has been approved in Japan for treating chronic pancreatitis and esophagitis since January 2006 [98]. Multiple promising studies establish that both cysteine protease cathepsin B and L (CatB/L) as well as TMPRSS2 are essential for the priming of S protein of SARS-CoV-2, Ebola, and MERS-CoV viruses in order to facilitate the fusion with host cells [99,100,101]. Recently, camostat mesylate was tested successfully to inhibit TMPRSS2 protease in vitro and blocks the entry of SARS-CoV-2 into the cells [99]. However, novel SARS-CoV-2 also utilizes CatB/L proteases for S priming, similar to SARS-CoV, and complete blockade of virus uptake is not attained with camostat mesylate. TMPRSS2 is an attractive target for therapeutic intervention against SARS-CoV-2 transmission and inhibition of TMPRSS2 did not impact the survival or development in a knockout mouse model [35]. The efficacy of camostat mesylate is being evaluated in three clinical trials for COVID-19 treatment.

Nafamostat mesylate is another serine protease inhibitor approved in Japan for treating pancreatitis since 1986 [102]. It acts as an anticoagulant [103] and has been shown to improve the disseminated intravascular coagulation associated with hematological disorders [104]. Another study examined that nafamostat mesylate moderately inhibited the membrane fusion of MERS-CoV in a dual spilt protein (DSP) based cell-cell fusion assay at a 10-time lower concentration than camostat mesylate [36]. Nafamostat mesylate is hypothesized to inhibit the TMPRSS2 protease. Hence, two clinical trials (phase 2/3) are underway to evaluate its safety and efficacy for COVID-19 treatment.

In addition to small molecules, protein-based serine protease inhibitors have also been reported to be effective against viral infections. Serpins, including serp-1 and serp-2 are derived from myxomavirus and exhibit immunomodulatory effects [105]. Serp-1 also improved the survival of mice infected with an unrelated virus-like mouse-adapted Zaire ebolavirus and murine γ-herpes virus 68 [106]. Recently, SERPINA1 (α1-antitrypsin or α1-AT) was identified to prevent the SARS-CoV-2 infection in vitro by specifically inhibiting the serine protease TMPRSS2 essential for the entry of virus [107]. α1-AT showed an IC_50_ of ~2mg/mL and comparable efficacy with camostat mesylate without any cytotoxic effects [108]. Hence α1-AT, an FDA approved drug, can be repurposed as a potential therapeutic intervention for COVID-19 treatment.

#### 2.1.6. Low Dose Heparins

Heparins are the most widely used anticoagulants that are clinically approved for prevention of pulmonary embolism, venous thrombosis as well as treatment of disseminated intravascular coagulopathies (DIC) [109]. Recently, a retrospective study reported a significant increase in D-dimer, fibrin degradation products, and features of DIC in 71.4% COVID-19 non-survivors [110]. Hence, alteration in the hematopoietic system is directly correlated with the severity of COVID-19 infection [111]. Further, the use of a low molecular weight heparin reduced mortality and improved the D-dimer levels in COVID-19 patients after 28 days [112]. In addition to its anticoagulant effect, heparin has pleiotropic effects including anti-inflammatory, anti-angiogenesis, antiviral, and anti-tumor [113]. Heparin is negatively charged glycosaminoglycan that insulates the cytokines to prevent their interaction with receptors and thus curtail the inflammatory response. Similarly, due to the polyanionic nature, heparin interacts with several positively charged proteins and prevents the entry of herpes simplex virus by competing for the cell surface glycoproteins [114]. Heparin (100 μg/mL) was also noted to inhibit the SARS-CoV infection by 50% in vero cells [115]. Recently, a computational study confirmed the interaction of heparin with basic amino acid patches on the surface of RBD on S1 subunit on spike protein of SARS-CoV-2, indicating the possibility of repurposing heparin for treatment of COVID-19 [116].

#### 2.1.7. Other Drugs

Nitazoxanide (Alinia®) is an FDA approved anti-protozoal drug. Both, nitazoxanide and its active metabolite tiazoxanide, possess broad-spectrum antiviral properties [117]. Nitazoxanide was found to be efficacious against Ebola in vitro [118] and influenza virus in a phase 2/3 clinical trial [119]. It also showed activity against MERS, SARS-CoV and other strains of HCoVs in vitro [37]. Its broad-spectrum activity is due to the interference with host immune response by inhibition of cytokine production [120]. Currently, it is being evaluated in five clinical trials for COVID-19 treatment.

Clevudine is a pyrimidine analog that exhibits antiviral efficacy by inhibiting the synthesis of the viral genome and has been approved for the treatment of a hepatitis B virus (HBV) in South Korea [38,39]. Currently, the safety and efficacy of clevudine are being examined in a phase 2 clinical trial. Favipiravir (T-705), a purine analog, is an inhibitor of RdRp approved in Japan for the treatment of influenza since 2014 [40]. T-705 showed potency against the Ebola virus in an in vivo model [121] as well as RNA viruses like rhinovirus or poliovirus [122]. Recent results from a clinical trial reported no significant improvement in T-705 treated group as compared to arbidol in COVID-19 patients [123]. To date, the safety and efficacy of T-705 are being evaluated in seven clinical trials against COVID-19.

Oseltamivir is a nucleoside analog and neuraminidase inhibitor that has been approved by the FDA for prophylaxis and treatment of influenza since 1999 [41,64]. Oseltamivir is also in clinical trials for COVID-19 treatment. So far, it did not show efficacy against SARS-CoV-2 in in vitro studies. Additionally, Oseltamivir was tested on few COVID-19 patients in China and no benefits were reported [124].

Darunavir/cobicistat (800 mg/150 mg) is an anti-HIV drug which has been approved by the FDA since 2015 [125]. Darunavir is a HIV protease inhibitor and cobicistat is a CYP3A inhibitor that improves the pharmacokinetic properties of darunavir [42]. Although a recent study shows darunavir’s low efficacy to inhibit SARS-CoV-2 in vitro [126], a clinical trial based on darunavir/cobicistat combination has been initiated in COVID-19 patients. Similarly, the combination of emtricitabine and tenofovir disoproxil, approved by the FDA in 2004, is another anti-HIV combination being tested for COVID-19 treatment [127].

Selinexor is the first selective inhibitor of nuclear export (SINE) approved for the treatment of myeloma by the FDA in 2019 [43]. Selinexor reversibly inhibits the nuclear transporter protein: exportin 1, also known as chromosome region maintenance 1 (CRM1), that blocks the export of mRNAs to the cytoplasm. SINE compounds have been extensively explored as a therapeutic intervention for multiple cancers [128] as well as viral infections [129]. Inhibition of CRM1 interferes with viral replication and protein synthesis that result in decreased viral activity. Antiviral efficiency of SINE compounds has been reported against HIV [130], influenza [131], Dengu [132], and Venezuelan equine encephalitis [133], just to name a few. Based on the aforementioned applications, 2 clinical trials are ongoing to test the efficacy and safety of selinexor for COVID-19.

Ivermectin, a broad-spectrum antiprotozoal drug, was approved by the FDA in 1996. Ivermectin also exhibits a broad-spectrum in vitro antiviral activity by blocking nuclear importin transporter (IMPα/β) [44] that inhibits the nucleo-cytoplasmic shuttling of the viral proteins [134]. Recently, ivermectin showed efficacy against SARS-CoV-2 (IC50 2 μM) in vitro [135] and has been hypothesized to show a synergistic effect in combination with HCQ [136]. MedinCell is developing a long-acting formulation of ivermectin for treatment of COVID-19 and 12 clinical trials are ongoing to test the efficacy of ivermectin against COVID-19.

Similarly, carrimycin, a macrolide antibiotic developed in China, is effective against gram-positive bacteria [45] and the efficacy is also being evaluated for the treatment of COVID-19. Ribavirin is another antiviral, approved for chronic hepatitis C infection by the FDA. Ribavirin is a guanosine analog, which acts as a direct antiviral via inhibiting the RdRp enzyme and destabilizing the viral RNA by interfering with natural guanosine and RNA capping. It also indirectly modulates the immune response by altering the expression of cytokines [137]. There are multiple studies reporting the efficacy of ribavirin against SARS-CoV [138] and MERS-CoV [139] in combination with corticosteroids. In a molecular docking study, ribavirin demonstrated binding to the RdRp of SARS-CoV-2 virus [46]. When tested in vitro in vero E6 cells, ribavirin showed an EC50 higher than 500 μM [64]. Besides, in a retrospective study, a combination of IFN-α and lopinavir/ritonavir with ribavirin was associated with favorable outcomes in COVID-19 patients [47]. Furthermore, the efficacy of ribavirin in combination with other anti-viral for COVID-19 is being tested in two randomized clinical trials.

### 2.2. Immunomodulators

The COVID-19 is associated with the stimulation of a cytokine storm that leads to elevation in inflammatory cytokines like interleukin (IL)-6, IL-2, IL-7, granulocyte colony-stimulating factor (GCSF), tumor necrosis factor-α (TNF-α) and monocyte chemoattractant protein 1 (MCP-1) in severely ill COVID-19 patients [124]. The inflammatory response elicited by COVID-19 results in acute respiratory distress syndrome (ARDS) and is responsible for high mortality rate. Hence, modulation of immune responses is another strategy to manage the severity of infection in COVID-19 patients. Several immunosuppressant drugs (Table 1) are being repurposed for treatment of COVID-19 patients and their efficacy is being evaluated in multiple clinical trials.

#### 2.2.1. Janus Kinase (JAK) Inhibitors

JAK is a janus kinase signal transducer which activates the transcription (JAK-STAT) signaling pathway that engages cytokines, interferons, and growth hormones to regulate the gene expression and cellular pathways [140]. Activation of JAK/STAT pathway induces the synthesis of inflammatory cytokines involved in autoimmune disorders, cancer, bacterial as well as viral infections [141].

Baricitinib is a JAK inhibitor that has been approved by the FDA for rheumatoid arthritis (RA) treatment. Baricitinib specifically inhibits JAK1 and JAK2, and results in the reduction of cytokines (IL-6) and immunoglobulins in the plasma of RA patients. Baricitinib also inhibits the numb associated kinase (NAK) that regulates clathrin-mediated endocytosis [142]. Recently, baricitinib has been hypothesized to exhibit direct antiviral activities by preventing the entry of the SARS-CoV-2 virus via inhibition of endocytosis pathway [48]. Hence, baricitinib is being evaluated for COVID-19 treatment in six clinical trials.

Ruxolitinib, a JAK inhibitor, was the first therapy approved in the US for myelofibrosis treatment [49]. Ruxolitinib has been repurposed for treating hospitalized COVID-19 patients to improve the survival by ameliorating the lung injury due to cytokine storm [143]. Eight clinical trials are ongoing for evaluating the efficacy of ruxolitinib to avert ARDS in COVID-19 patients.

#### 2.2.2. Fingolimod

Fingolimod is a small molecule modulator of sphingosine-1-phosphate receptor subtype 1 and has been approved for treatment of multiple sclerosis by the FDA [50]. Fingolimod induces the release of lymphocytes from the lymphoid organs and reduces the inflammatory response [144]. Currently, fingolimod is in a phase 2 clinical trial to regulate the immune response in severely infected COVID-19 patients.

#### 2.2.3. Aviptadil

Aviptadil, a combination of synthetic vasoactive intestinal polypeptide (VIP) (~28 amino acids) and phentolamine, is approved in Europe for the treatment of erectile dysfunction [51]. The VIP is a neuropeptide mostly present in the lungs and nasal mucosa. Aviptadil elicits potent bronchodilation, vasodilation, and anti-inflammatory effects, useful for management of respiratory disorders, such as asthma, cystic fibrosis, sarcoidosis, and pulmonary arterial hypertension (PAH) [145]. Based on the bronchodilatory and anti-inflammatory effects, intravenous aviptadil is being evaluated in two phase 2 clinical trials for the management of ARDS associated with COVID-19.

#### 2.2.4. Thalidomide

Thalidomide, in combination with glucocorticoid, was reported to successfully treat a COVID-19 patient in China [146]. Since the withdrawal of thalidomide from the market due to teratogenic effects, it has been repurposed as an anti-cancer and anti-inflammatory drug [147]. It improved the survival of BALB/c mice after exposure to H1N1 virus by reducing the cytokine levels (IL-6, TNF-α) [52]. Similarly, reduced cytokine levels were observed in the patient that recovered from COVID-19 after treatment with the combination of thalidomide and glucocorticoid. These promising results warranted the initiation of two phase 2 clinical trials to test the efficacy of thalidomide for treatment of COVID-19.

#### 2.2.5. Monoclonal Antibodies (mAbs)

About 24 clinical trials are ongoing where the efficacy of different monoclonal antibodies is being tested for treatment of COVID-19. The elevated levels of cytokines IL-7, IL-10, IL-6, and IFN-α are indicative of severe inflammatory response and cytokine storm in COVID-19 patients [148]. Among these, IL-6 and granulocyte-macrophage colony-stimulating factor (GM-CSF) are the key cytokines responsible for this dysregulated inflammatory response. Hence mAbs targeting IL-6 receptors (tocilizumab and sarilumab) and GM-CSF (lenzilumab, an investigational drug) have been repurposed for treating COVID-19 patients.

Tocilizumab, an IL-6 receptor antagonist, is indicated for rheumatoid arthritis [149] and cytokine release syndrome [150]. In a recent study, tocilizumab was reported to significantly improve the clinical outcome in critical COVID-19 patients (*n* = 19), with all patients showing improvement within 15 days [53]. Similarly, positive outcomes with tocilizumab therapy were reported in another retrospective study in COVID-19 patients [150]. In addition, 18 clinical trials are ongoing to test the safety and efficacy of tocilizumab for the treatment of COVID-19. Moreover, sarilumab, which is the second IL-6 receptor antagonist, approved for the treatment of RA [151], also entered a phase 3 clinical trial for COVID-19.

Bevacizumab is a vascular endothelial growth factor (VEGF) inhibitor approved for the treatment of tumors like colorectal and glioblastoma by the FDA [152]. VEGF induces angiogenesis and bronchodilation, enhances vascular permeability, and promotes tumor growth [153]. The role of VEGF in lung injury or ARDS is controversial, but a recent study reported that high VEGF levels were responsible for fat embolism-induced ARDS [54]. Bevacizumab’s efficacy is being evaluated in two phase 2/3 clinical trials for treating ARDS in critical COVID-19 patients.

Eculizumab, approved for the treatment of paroxysmal nocturnal hemoglobinuria, targets the complement protein C5 to regulate inflammatory response [55]. 3 clinical trials are ongoing to test eculizumab for COVID-19 treatment. 

#### 2.2.6. Interferons

Type I interferons (IFN-1: IFNα and IFNβ), also known as viral IFNs, are cytokines, which trigger antiviral immune responses. IFNs have been approved for the treatment of multiple sclerosis [154]. IFN-1 activates the production of interferon-stimulated genes (ISG) that mediates signaling and immunomodulation [155]. IFN-1 treatment was studied against SARS-CoV with or without antiviral drugs in vitro and in SARS-CoV patients [156]. Furthermore, IFNβ has been reported to be more effective in comparison to IFNα against SARS-CoV [157] and MERS-CoV [158]. A recent study indicated that SARS-CoV-2 is sensitive to IFN-1 pretreatment as it lacks the ORF3b and has a modified ORF6 that is required to inhibit IFN-1 [159]. Recently, IFNα2b showed efficacy in reducing SARS-CoV-2 viral load when used prophylactically via inhalation [160]. The IFN-1 therapy is suggested only for the early stages of the infection as excessive activation via IFN-1 might contribute to a cytokine storm [56]. A retrospective study stated that treatment of COVID-19 patients with IFNα2b reduced the viral load and levels of inflammatory cytokine IL-6 with or without arbidol [57]. Recently, the results of a phase 2 randomized clinical trial indicate expedited clinical improvement in COVID-19 patients with early symptoms after treatment with IFNβ1b in combination with lopinavir/ritonavir/ribavirin in comparison to only lopinavir/ritonavir/ribavirin group [161].

IFNλ, type III interferon, is another cytokine that exhibits antiviral activity at epithelial surfaces and prevents excessive systemic inflammatory response [162]. The safety and efficacy of pegylated IFNλ are being tested in a phase 2 clinical trial (NCT02765802) for the treatment of hepatitis. IFNλ therapies are in clinical trials for a myriad of disorders and their efficacy either alone or in combination with antivirals is also being tested for COVID-19 in 18 clinical trials.

### 2.3. Investigational Drugs

#### 2.3.1. Remdesivir

Remdesivir (RDV or GS-5734), an adenosine nucleotide analog (Table 2) and an investigational drug for treatment of Ebola virus, acts by inhibiting the RdRp enzyme essential for replication of RNA viruses [163]. The phase 2 clinical trial results of RDV for the treatment of Ebola virus were not promising [164]. However, it has shown efficacy against coronavirus strains like HCoV-NL63, MERS-CoV, bat-CoV, as well as SARS-CoV-2 in human airway epithelial cells in vitro [165]. Besides, RDV was reported to be effective for prophylaxis as well as treatment of SARS-CoV infection in a mouse model. RDV has also shown efficacy in mice [166] and non-human primate models (rhesus macaque) of MERS-CoV [167] and SARS-CoV-2 [168].

A recent study found that the triphosphate form of RDV can potentially compete with ATP and after incorporation in the RNA sequence at position (I), it inhibits the RNA synthesis at (I + 3) position [169]. In initial screenings of drugs against SARS-CoV-2, RDV inhibited the virus in vitro at low micromolar concentrations with EC50 of 0.77 μM [73]. A cohort study reported that 68% of severe COVID-19 patients (*n* = 53) showed clinical improvement after 10 days of treatment with RDV [170]. Based on the evidence, the efficacy of RDV is being tested for COVID-19 treatment in 10 phase 2/3 clinical trials. Recently, promising results from a randomized clinical trial of RDV were reported, where patients receiving (*n* = 158) RDV showed faster clinical improvement in comparison to the placebo group [171]. Based on these results, the FDA issued an EUA to remdesivir (GS-5834TM) for treatment of severe COVID-19 patients on May 1st, 2020.

#### 2.3.2. Tradipitant

Tradipitant, an inhibitor of neurokinin-1 (NK1) receptor, is being investigated in phase 2/3 clinical trials for acute dermatitis, gastroparesis, and motion sickness. NK1 acts as a receptor for neuropeptide substance P and plays a role in neuroinflammatory disorders. In addition to CNS, NK1 receptor is also present in the gastrointestinal tract, skin, and lungs [172]. One study reported the role of NK1 receptor in exacerbating inflammatory response in rat lungs after infection with a respiratory syncytial virus (RSV) [173]. Hence, tradipitant is being evaluated in a phase 3 clinical trial to minimize the neurogenic inflammation in the lungs of COVID-19 patients.

#### 2.3.3. ASC09 (TMC 310911)

ASC09 or TMC 310911 is an investigational HIV-1 protease inhibitor (PI) and has been reported to be effective against multiple PI resistant HIV strains [174]. The efficacy has also been established in HIV patients in a phase 2 clinical trial [180] and currently it is being tested in a phase 3 clinical trial for COVID-19 treatment.

#### 2.3.4. FT516

FT516 is the first natural killer cell (NK) based cancer immunotherapy developed by Fate Therapeutics (La Jolla, CA, USA). FT516 is a genetically engineered NK cell-expressing high-affinity non-cleavable CD16 Fc receptor, derived from modified induced pluripotent stem cells (iPSC) that exhibits superior efficacy against tumor cells via antibody-dependent cellular cytotoxicity (ADCC) pathway. NK cells are essential for ADCC mediated immune response where they can recognize and eliminate the antibody (IgG) coated virus-infected cells. Hence FT516 can result in enhanced NK-mediated immune response against SARS-CoV-2 infection [175] via anti-COVID-IgG antibodies. It is currently being tested in a phase 1 clinical trial for hospitalized COVID-19 patients with hypoxia.

#### 2.3.5. CD24Fc

CD24Fc is an immunomodulator currently being evaluated in a phase 2/3 clinical trial (NCT02663622) for graft versus host disease in patients after the transplant of hematopoietic stem cells. CD24Fc acts as an immune checkpoint for inflammatory response mediated by damage-associated molecular patterns (DAMP) activated during viral infections [176]. A recent study found that CD24Fc improved survival and ameliorated lung damage in simian immunodeficiency virus (SIV)-infected rhesus monkey [177]. Hence CD24Fc is being tested as an immunomodulator drug in COVID-19 patients in a phase 3 clinical trial (NCT04317040).

#### 2.3.6. Others

XPro1595, a TNF inhibitor, is being investigated for the treatment of Alzheimer's disease in a phase 1 study (NCT03943264). Since TNF is an inflammatory cytokine, whose levels are elevated in COVID-19, it is being tested for management of pulmonary complications in COVID-19 patients in a phase 2 clinical trial. LY3127804 is an antibody against angiopoietin-2 and is being tested in a phase 1 clinical trial for the treatment of solid metastatic tumors. Angiopoietin 2, an inflammatory cytokine, plays a pathological role in inflammation and lung injury associated with pneumonia [178]. Hence, the efficacy of LY3127804 is being evaluated in COVID-19 patients with pneumonia in a phase 2 clinical trial.

Leronlimab or PRO 140 is an investigational C-C chemokine receptor type 5 (CCR5) antibody found to be efficacious in a phase 3 clinical trial for the treatment of R5 HIV patients [181]. A recent study reported that leronlimab reduced the inflammation, plasma viremia levels, and T cell lymphocytopenia via blocking the CCL5/RANTES-CCR5 pathway in COVID-19 patients [179]. Two clinical trials (phase 2b/3) are on-going to test the efficacy of leronlimab for the treatment of COVID-19.

### 2.4. Convalescent Plasma Therapy (CPT)

In CPT, donated blood from the patients who have fully recovered from the COVID-19 is used as a treatment for patients still battling the illness, since recovered patients have developed antibodies against SARS-CoV-2 infection [182]. Here, the blood collected from the COVID-19 patients after 14 days of recovery undergoes apheresis to separate plasma containing neutralizing antibodies against SARS-CoV-2 and anti-inflammatory cytokines, defensins, pentraxins, or clotting factors [183]. CPT has been widely used for post-exposure prophylaxis or treatment during previous outbreaks [184,185]. Based on the previous success, CPT has been used extensively for the treatment of COVID-19 patients and is known to be the most effective strategy for prevention and treatment in early stages of the infection [186].

Although the SARS-CoV-2 vaccine will ultimately be the first preventative choice, CPT can be used for now to provide immediate short-term immunity to patients until vaccines are developed and approved for use. In addition to direct neutralization of the virus by anti-SARS-CoV-2 antibodies, CPT provides enhanced protection via complement activation, ADCC, or phagocytosis [186]. In a case report, convalescent plasma with an antibody titer of 1:1000 (400 mL) improved the clinical symptoms of 5 severely infected COVID-19 patients [187]. Another study showed improvement in symptoms of COVID-19 patients within three days of administering convalescent plasma with an antibody titer of 1:650 or more (200 mL) [188]. A recent systematic review, including five studies on the use of CPTs in COVID-19 patients, concluded it as a safe and effective therapeutic intervention that reduced the patient mortality rate [189]. However, CPT based treatment requires further optimization, including timing to start the therapy, plasma volume, minimum antibody titer, impact of concomitant use of antivirals and corticosteroids, just to name a few, for its broader clinical utility. In addition, certain associated risks like allergic transfusion, viral transmission, circulatory overload, and transfusion-related acute lung injury need to be studied in large-scale clinical trials [190]. To date, 69 clinical trials are registered to evaluate the efficacy of CPT in COVID-19 patients.

### 2.5. SARS-CoV-2 Vaccine Candidates

**Figure 4:**
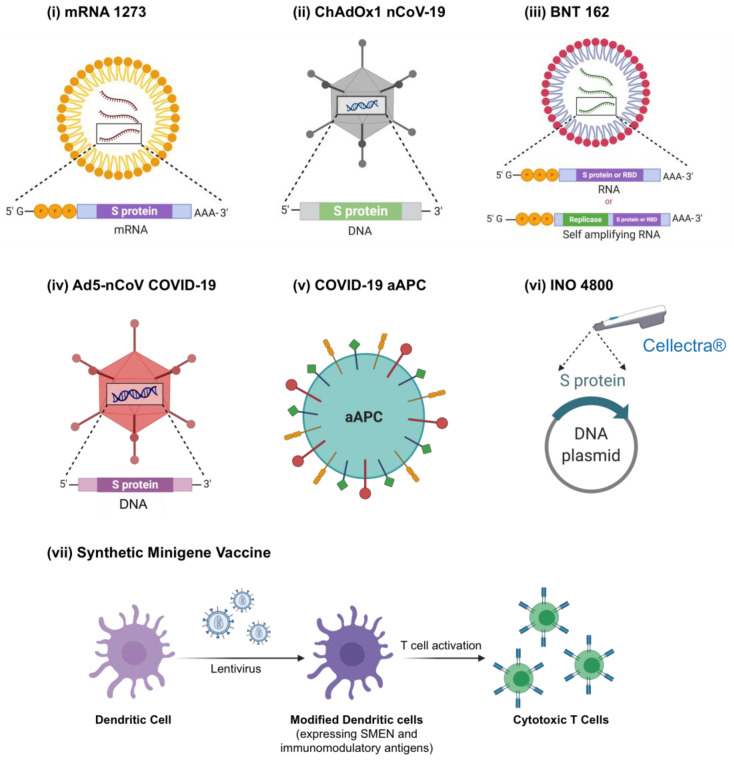
Vaccine candidates currently in clinical trials for COVID-19. (**i**) mRNA 1273 is a mRNA-based vaccine, which encodes the full-length S protein of SARS-CoV-2 virus and is delivered via lipid nanoparticles (LNPs). (**ii**) ChAdOx1 nCoV-19 is a chimpanzee adenovirus vector, which expresses the S protein of SARS-CoV-2 virus inside the host cells and activates the immune system. (**iii**) BNT 162 is a mRNA-based vaccine delivered via LNPs with four candidates (BNT162a1, BNT162b1, BNT162b2, BNT162c2), encoding either the S protein or receptor binding domain (RBD) of S1 subunit. (**iv**) Ad5-nCoV COVID-19 is a replication defective adenovirus 5 vector (Ad5) encoding the full-length S protein of SARS-CoV-2 virus. (**v**) COVID-19 aAPC are artificial antigen-presenting cells (aAPC) modified using a lentivirus vector to express fragments of SARS-CoV-2 proteins and immunomodulatory genes. (**vi**) INO 4800 is a plasmid DNA encoding SARS-CoV-2 proteins and delivered via electroporation using the smart device Cellectra® developed by Inovio. (**vii**) Synthetic Minigene Vaccine or LV-SMENP-DC are genetically modified dendritic cells (DCs) via a lentivirus vector to express SARS-CoV-2 minigenes (SMEN) and immunomodulatory genes, administered via subcutaneous injection. Furthermore, T cells activated using the modified dendritic cells are also administered via intravenous infusion.

The rapid development of a vaccine against SARS-CoV-2 is imperative to achieve herd immunity without suffering high mortalities due to multiple waves of infection. The world has witnessed an unprecedented and accelerated vaccine development at a pace never before seen in modern medicine. The first vaccine candidate (mRNA1273, Moderna, Cambridge, MA, USA) against SARS-CoV-2 entered clinical trials on March 16, 2020, only two months after the genome sequence of the novel SARS-CoV-2 virus was published on January 11, 2020 [191]. The landscape of COVID-19 vaccines, published by the WHO on May 5, 2020, reported that more than 100 vaccine candidates are at the pre-clinical stages and seven candidates are in clinical trials. The vaccine candidates registered by the National Institutes of Health (NIH) (clinicaltrials.gov) are discussed below (Figure 4) and summarized in Table 3.

It is notable that there are currently a large number of highly experimental vaccination approaches under development that utilize non-traditional methods to express immunizing antigens in patients. These emerging approaches include directly administering mRNAs to cause antigen expression in vivo, as well as introduction of antigen-presenting viruses and DNA constructs. It remains to be seen if these novel approaches will have the same efficacy and strong safety as has been found with traditional protein antigen- and attenuated viral vaccines.

#### 2.5.1. mRNA-Based Vaccines

mRNA-1273, developed by Moderna, is a lipid nanoparticle (LNP) delivered mRNA encoding the S-2P antigen of SARS-CoV-2 S glycoprotein consisting of S1-S2 cleavage site [192]. As explained in the introduction, the S protein interacts with the human ACE2 enzyme and is essential for viral entry in the host via endocytosis, making it an attractive target for vaccine development [16]. The mRNA coding for the S-protein is transcribed in vitro and contains 5’ methylation and 3’ poly-A chain in the untranslated regions at 5’- and 3’-ends to enhance its stability and promote translation inside the cytoplasm [193]. The mRNA is encapsulated in LNPs to overcome the limitations like poor stability, low cellular uptake, and to reduce the toxicity and immunogenicity [194]. mRNA-1273-loaded LNPs are administered intramuscularly and undergo uptake into local myocytes via endocytosis to release mRNA in the cytoplasm where it undergoes translation to form the S protein. The translated S protein is then secreted outside the cells and activates both the cellular and humoral immune response resulting in active immunization against the SARS-CoV-2 virus [195]. mRNA-based vaccines exhibit several advantages over traditional approaches, including low risk of mutagenesis [196], high potency resulting in one or two immunizations [197], high translation efficacy due to structural modifications [198], and rapid large-scale production. Recently the interim results of a phase 1 dose range study (NCT04283461) were published where two vaccinations of either 25 μg, 100 μg, or 250 μg, administered 28 days apart, were tested in healthy adults [199]. The study reported a significant increase in anti-IgG antibody against S-2P subunit after 14 days and showed dose-dependent increase in the antibody response. Based on these results, a phase 3 study (NCT04470427) to evaluate the efficacy, safety and, immunogenicity of the mRNA-1273 vaccine has been initiated.

BNT162 is another mRNA-based vaccine developed by Pfizer (NY, USA) and BioNTech (Mainz, Germany). BioNTech has expertise in developing mRNA-based platform/therapeutics for the treatment of cancer, rare genetic disorders, and infectious diseases. In collaboration with Pfizer, they have developed four mRNA vaccine candidates against the SARS-CoV-2 infection. These candidates are based on versatile mRNA therapeutics’ platform; optimized unmodified mRNA (uRNA), nucleoside modified mRNA (modRNA), and self-amplifying RNA (saRNA). All the mRNA vaccine candidates encode for either the full S-protein or RBD domain of the S1 subunit, which interacts with the ACE2 enzyme and is encapsulated in LNPs for improved intracellular delivery. In prior studies, saRNAs derived from alphaviruses contain two ORFs, where one encodes for the enzyme replicase, RdRp and another for the target antigen [200]. Hence, a saRNA results in multiple copies of target antigenic RNA mediated by replicase and was reported to induce robust immunogenic response at a much lower dose against influenza in vivo [201]. Recently, a large-scale phase 1/2 clinical trial (NCT04368728) to evaluate the safety, immunogenicity, optimum dose, and efficacy of mRNA-based vaccine candidates was completed. The preliminary results from the study indicated superior tolerability profile and T-cell response against RBD as well as other parts of spike protein by BNT162b2, a modRNA based vaccine, at 30 μg dose in a two-dose regimen [202]. Although the complete results of the study are yet to be published, however, a phase 2/3 clinical trial is currently being planned to test the efficacy of BNT162b2 in up to 30,000 participants globally.

#### 2.5.2. INO 4800 DNA Vaccine

INO 4800, developed by Inovio (Plymouth, PA, USA), consists of a plasmid DNA coding for SARS-CoV-2 proteins and is administered intradermally via electroporation. Cellectra®, a smart device developed by Inovio, uses electroporation that creates transient pores in the skin to allow the penetration of DNA vaccine [209]. The safety and efficacy of Cellectra® as a delivery platform for a DNA vaccine or immunization via an intramuscular or intradermal route were established in an open label study in healthy volunteers [210]. Plasmids, similar to virus, utilize host cell machinery to express the antigenic proteins in the cytoplasm, resulting in activation of both cellular and humoral immune response [211]. Recently, the results of a phase 1 study (NCT04336410) were announced by Inovio, where 94% of the enrolled adults showed an immunological response, T-cell mediated as well as humoral, against SARS-CoV-2 at both the 1.0 mg and 2.0 mg dose administered twice at 4 weeks interval without any severe adverse effects [208]. Further, the company is planning a phase 3 study to evaluate the efficacy of INO 4800 against SARS-CoV-2. INO 4800 is the only vaccine candidate stable at room temperature for one year and hence, will fast-track the mass immunization when approved by the FDA.

#### 2.5.3. ChAdOx1 nCoV-19 Vaccine

ChAdOx1, a chimpanzee adenovirus vector derived from Y25 isolate of chimpanzee adenovirus, has shown a low immunologic response in human serum samples [212]. In a recent report, ChAdOx1 MERS capable of expressing the S protein of MERS-CoV was found to be safe and tolerated even at a high dose of 5 × 10^10^ viral particles with minimum side effects in a phase 1 clinical trial [213]. It has also been reported to be effective as a vector to provide immunization against hepatitis B virus in mice [203]. Similarly, ChAdOX1 nCoV-19 encodes the S glycoprotein of novel SARS-CoV-2. The safety, efficacy, and immunogenicity of ChAdOX1 nCoV-19 was evaluated in healthy adults at a dose of 5 × 10^10^ viral particles (NCT04324606) [204]. The results from the study demonstrated that a single dose of vaccine elicited the anti-spike IgG response after 28 days which boosted with a second dose. Further, administration of the vaccine also induced cell-mediated immune response and showed an acceptable safety profile. Currently, the efficacy of ChAdOX1 nCoV-19 is being evaluated in a large scale phase 3 clinical trial (NCT04400838) against COVID-19.

#### 2.5.4. COVID-19 Artificial Antigen-Presenting Cells (aAPC) Vaccine

aAPC are superior to dendritic cells for activating the cytotoxic T-cells against the specific antigen [214]. This approach is extensively being explored for cancer immunotherapy [215]. COVID-19 aAPC vaccine, developed by China’s Shenzhen Geno-Immune Medical Institute (GIMI), utilizes lentivirus vector to introduce gene fragments coding different SARS-CoV-2 proteins as well as immunomodulatory genes into the aAPC and hence, activates the immune system upon subcutaneous injection. Lentivirus vectors (LVs) are capable of invading the host genome of non-dividing cells and guide the production of encoded genes [216]. LVs have been extensively used to modify the immune cells for cancer immunotherapy [205] as well as hematopoietic stem cells (HSC) for genetic disorders [206]. The safety and efficacy of COVID-19 aAPC vaccine are being tested in a phase 1 clinical trial (NCT04299724) in COVID-19 positive and healthy volunteers. 

#### 2.5.5. Synthetic Minigene Vaccine

Synthetic minigene vaccine is another vaccine candidate, developed at GIMI (Shenzhen, China) by genetically modifying the dendritic cells (DCs) to express SARS-COV-2 minigenes (SMEN) as well as immunomodulatory genes using a lentivirus vector and production of antigen specific cytotoxic T cells (CTLs) on exposure to modified DCs (Figure 4). The combination vaccine (5 × 10^6^ LV-DC vaccine subcutaneous injection + 1 × 10^8^ CTLs intravenous infusion) is being tested in COVID-19 patients in a phase 1/2 clinical trial (NCT04276896).

#### 2.5.6. Ad5-nCoV COVID-19 Vaccine

Ad5-nCoV COVID-19 vaccine utilizes a recombinant replication defective adenovirus 5 vector (Ad5) which encodes for the full S-glycoprotein of SARS-CoV-2. Ad5 vector has been extensively studied for gene therapy-based applications and safety has been established in multiple clinical trials [217]. However, in a phase 2 study, the Ad5-based HIV vaccine did not prevent the HIV infection in male subjects [218]. The safety and tolerance of Ad5-EBOV, another Ad5 based vaccine against Ebola, was established in clinical trials [219] and was later approved in China. The results of the first-in-human clinical trial (NCT04313127) to determine the safety, tolerability and immunogenicity of Ad5-nCoV COVID-19 vaccine were published on May 22, 2020. The study conducted at three different doses reported a peak of neutralizing antibodies at 28 days post-vaccination and T-cell mediated response within 14 days in healthy adults [207]. Based on these results, another phase 2 study (NCT04341389) reported significant immune response and safety at a dose of 5 × 10^5^ viral particles [220]. Hence, a large-scale phase 3 study is being planned to test the effectiveness of the vaccine.

### 2.6. Mesenchymal Stem Cell (MSC) Therapy

MSCs are multipotent cells capable of division and differentiation and are derived from either blood, umbilical cord, bone marrow, or adipose tissue to be used for regenerative or immunomodulatory purposes in a wide range of disorders like hematopoietic stem cell transplantation for B cell malignancies and genetic disorders [221]. MSCs have been found to be efficacious in alleviating the ARDS in severe COVID-19 patients [222]. Besides, another study reported that 7 COVID-19 patients treated with MSCs showed significant improvement in symptoms within 2 days of treatment, while increase in lymphocytes, decrease in cytokine-secreting immune cells and TNFα was observed after 3–5 days [223]. Further studies revealed that MSCs lacked ACE2 and TMPRSS2, which are essential for entry of SARS-CoV-2, indicating that MSCs are immune to infection. After intravenous administration, MSCs are known to accumulate in lungs where they can proliferate and reduce the lung injury, while protecting the alveolar epithelial cells. In addition to stem cell activity, MSCs can also act as an immunosuppressant in the presence of an inflammatory environment [224]. Currently, the efficacy of MSCs therapy is being tested in 15 clinical trials to treat COVID-19 patients.

## 3. Emerging Therapeutics

In addition to the on-going clinical trials, several therapeutic strategies are currently being explored or tested at pre-clinical stages to combat SARS-CoV-2. Microneedle arrays (MNAs) based on water-soluble polymers have been established as a minimally invasive approach for intra-cutaneous delivery of vaccines [225,226]. Recently safety and immunogenicity of microneedle array (MNA) delivered mRNA encoding spike glycoprotein’s S1 subunit of SARS-CoV-2 and MERS-CoV was established in a mice model [227]. The codon for S1 subunit of SARS-CoV-2 and MERS-CoV was fused with a sequence of a foldon domain from bacteriophage T4 fibritin [228,229] to mimic the native trimeric structural arrangement of the virus. Further, the sequences were integrated with RS09, a toll-like receptor-4 agonist (TLR4), to augment the activation of the inflammatory response [230]. The study reported a significant antibody response in mice after 6 weeks of vaccination.

Goal et al, tested the efficacy of PiCoVacc, an inactivated SARS-CoV-2 virus-based vaccine, in Wister rats, BALB/C mice, and rhesus macaques [231]. PiCoVacc was derived from the CN2 strain of SARS-CoV-2 collected from lavage samples of patients from China. The peak titers were detected in mice 6 weeks post-immunization at >100 μg/mL and >200 μg/mL for IgG antibodies against RBD and spike protein, respectively. Similar results were observed in Wistar rats. Further, three doses were administered intramuscularly in macaques at 0, 7, and 14 days, and antibodies reached peak at week 3. In a challenge study where 10^6^ TCID_50_ of SARS-CoV-2 CN1 strain were administered intratracheally, vaccinated macaques showed minimal histopathological changes in the lungs in comparison to the control group. Moreover, macaques that received a higher dose (6 μg) showed undetectable viral loads after 7 days. The safety of PiCoVacc was also established in macaques via multiple endpoints including biochemical, hematological and histopathological analysis.

## 4. Conclusions

In the present review, we provide a thorough overview of the current therapeutic modalities being tested in clinical trials for COVID-19 treatment. The contribution from the scientific community, pharmaceutical sector, healthcare agencies and governments across the globe, as well as the WHO, has been unprecedented in accelerating possible COVID-19 therapies, especially vaccine candidates to clinical trials. Among the therapeutic interventions discussed here, the WHO selected four of the most promising approaches to test in clinical trials at a global scale with participations from over 100 countries to determine the efficacy for treatment of COVID-19. The candidates, selected based on the promising data, include remdesivir, lopinavir/ritonavir, INFβ1a and chloroquine/ hydroxychloroquine, are being tested using the same standardized protocol across the global facilities to gather concrete evidence to tackle the pandemic. However, due to lack of efficacy in preliminary studies, treatment arms including lopinavir/ritonavir and chloroquine/ hydroxychloroquine were discontinued recently. In addition to the current therapies in clinical trials, there are a plethora of novel therapeutic modalities at multiple stages of development. However, even with today’s technological advances and resources, one novel strain of virus has brought the world to a standstill, indicating that we are far away from ready to handle a pandemic of this scale. By catching the global healthcare system unaware and unprepared, this pandemic has brought spotlight to the value of drug repurposing and the need for novel and faster approaches in drug development, bringing the researchers and policy makers together to better prepare for future outbreaks.

## Figures and Tables

**Table 1:** Description of repurposed drugs currently in clinical trials for COVID-19.

Drug(s)	Antiviral Activity	Number of Clinical Trials	Reference
Lopinavir/ritonavir	Coronavirus protease (CLpro) inhibitor	18	[26,27]
Chloroquine/ hydroxy-chloroquine	i. Acidification of endosomes/lysosomes ii. Impair glycosylation of angiotensin converting enzyme 2 (ACE2)	117	[28,29,30]
Azithromycin	i. Endosome acidification ii. Immunomodulatory	34	[31] [32]
Arbidol	i. Interference with virus trafficking ii. Blocks trimerization of spike (S) glycoprotein	3	[33] [34]
Camostat mesylate	Serine protease (TMPRSS2) inhibitor	3	[35]
Nafamostat mesylate	Serine protease (TMPRSS2) inhibitor	2	[36]
Nitazoxanide	Inhibition of cytokine production	5	[37]
Clevudine	Inhibition of viral RNA synthesis	1	[38,39]
Favipiravir	RNA-dependent RNA polymerase (RdRp) inhibitor	7	[40]
Oseltamivir	Neuraminidase inhibitor	4	[41]
Darunavir/cobicistat	HIV protease inhibitor/ CYP3A inhibitor	1	[42]
Selinexor	Selective inhibitor nuclear export (SINE)	2	[43]
Ivermectin	Inhibition of nuclear importin (IMPα/β) transporter	12	[44]
Carrimycin	Prevent respiratory tract infection	1	[45]
Ribavirin	i. RdRp inhibitor ii. Immunomodulatory	2	[46,47]
*Immunomodulators*			
Baricitinib	Janus kinase (JAK1 and JAK2) inhibitor	6	[48]
Ruxolitinib	Janus kinase inhibitor	8	[49]
Fingolimod	Anti-inflammatory (sphingosine-1-phosphate receptor subtype 1 modulatory)	1	[50]
Aviptadil	i. Broncho- and vasodilation ii. Anti-inflammatory	2	[51]
Thalidomide	Anti-inflammatory	2	[52]
Tocilizumab	IL-6 antagonist	18	[53]
Bevacizumab	Vascular endothelial growth factor (VEGF) inhibitor	2	[54]
Eculizumab	Complement protein C5 inhibitor	3	[55]
Interferons	Immune activation against virus	18	[56,57]

**Table 2:** List of investigational drugs currently being tested for COVID-19.

Drugs	Antiviral Activity	Number of Clinical Trials	Reference
Remdesivir	RdRp inhibitor	10	[171]
Tradipitant	Neurokinin-1 (NK1) antagonist	1	[173]
ASC09 or TMC 310911	Human immunodeficiency virus-1 (HIV-1) protease inhibitor	1	[174]
FT516	i. Genetically modified natural killer (NK) cells ii. Enhances cytotoxic response against SARS-CoV-2	1	[175]
CD24Fc	Immune check point and prevents hyperinflammation	1	[176,177]
XPro1595	Tumor necrosis factor (TNF) inhibitor	1	-
*LY3127804*	Angiopoietin-2 antibody	1	[178]
Leronlimab	C-C chemokine receptor type 5 (CCR5) antibody	2	[179]

**Table 3:** Description of SARS-COV-2 vaccine candidates currently in clinical trials.

Vaccine	Antiviral Activity	Clinical Trial	Reference
mRNA1273	mRNA based vaccine candidate encoding for complete S protein of SARS-CoV-2 Delivery platform: lipid nanoparticles (LNP)	NCT04470427 Phase 3 Status: Recruiting	[192,199]
BNT162	4 mRNA-based vaccine candidates encoding either - full length S glycoprotein of SARS-CoV-2 - Receptor binding domain (RBD) of S1 subunit of mRNA modifications: - optimized unmodified mRNA (uRNA), - nucleoside modified mRNA (modRNA) and, - self ampliflying RNA (saRNA) Delivery platform: LNPs	NCT04368728 Phase 1/2 Status: Active, Not Recruiting	[201,202]
ChAdOx1 nCoV-19	Chimpanzee adenovirus vector encoding S glycoprotein of SARS-CoV-2	NCT04400838 Phase 2/3 Status: Recruiting	[203,204]
COVID-19 aAPC	Artificial antigen-presenting cells (aAPC) modified using lentivirus vector to express - multiple fragments of SARS-CoV proteins - immunomodulatory genes	NCT04299724 Phase 1 Status: Recruiting	[205,206]
Synthetic Minigene Vaccine or LV-SMENP-DC	i. Genetically modified dendritic cells (DCs) via lentivirus vector to express - SARS-CoV-2 minigenes (SMEN) - Immunomodulatory genes ii. Cytotoxic T cells (CTCs) developed against modified DCs	NCT04276896 Phase 1/2 Status: Recruiting	-
Ad5-nCoV COVID-19	Replication defective adenovirus 5 vector (Ad5) encoding full length S protein of SARS-coV-2 virus	NCT04398147 Phase 2 Status: Active, Not Recruiting	[207]
INO 4800	Plasmid DNA encoding SARS-CoV-2 proteins Delivery platform: Cellectra^®^ (electroporation)	NCT04336410 Phase 1 Status: Recruiting NCT04447781 Phase I/IIa Status: Not yet recruiting	[208]
